# Promoting the Cause of Higher Medical Education

**Published:** 1888-03

**Authors:** 


Promoting the Cause of Higher Medical Education.—California has followed other states in refusing to recognize medical diplomas issued by colleges requiring attendance at only two courses of lectures before graduation. The following is the text of the resolution adopted: "On and after April 1, 1891, the Board of Examiners of the Medical Society of the State of California will not grant certificates to practice medicine on diplomas, issued after that date, by colleges which do not require that all candidates for graduation shall have studied not less than three full years, and shall have attended not less than three full regular courses of lectures during three separate years."

---

We call attention to the advertisement, in another column, of "The Language of Medicine," by our associate, Dr. F. R. Campbell. This is the only work in the English language dealing exclusively with the history, etymology and pronunciation of the technical terms employed in medical literature, and will consequently be of great interest to a large portion of the profession. The author's knowledge of the ancient and modern languages renders him eminently fitted for the composition of such a work, and we prophesy an extensive sale.

---

Two deaths have occurred during the past month in the profession of this city: Dr. J. E. King, an old and respected practitioner of forty years' standing, died suddenly, February 1st, and Dr. Chas. G. Steele, Secretary of the Buffalo Medical and Surgical Association, died of peritonitis, February 11th. Dr. Steele was but twenty-seven years of age, and had just entered upon the practice of his profession.

